# P067: Economic burden of methicillin-resistant Staphylococcus aureus, Clostridium difficile, and vancomycin-resistant enterococci in hospitals

**DOI:** 10.1186/2047-2994-2-S1-P67

**Published:** 2013-06-20

**Authors:** A Mitchell, R Rohde, P Mallow, D Buskirk

**Affiliations:** 1 Johnson & Johnson, Irvine, USA; 2 Texas State University, San Marcos, USA; 3 S2, Irvine, USA

## Introduction

Methicillin-Resistant *Staphylococcus aureus* (MRSA), *Clostridium difficile* (C. Diff) and Vancomycin-Resistant Enterococci (VRE) are a significant source of HAIs. Recently, significant attention has been given to community acquired strains of these infections. However, HAIs with these pathogens remain a significant cause of infections and associated costs to the health system.

## Objectives

The purpose of this study is to estimate economic burden of MRSA, CDI, and VRE HAIs among inpatients in US acute care hospitals.

## Methods

The economic burden of MRSA, C. Diff, and VRE were calculated based on incidence rates and costs attributable to the pathogen rates from the published literature. Studies included were those that estimated national incident rates of one or more of the HAIs. Cost estimates were used from studies that specifically stated direct costs associated with the HAIs. The median incidence rate and cost were derived for the base estimate.

## Results

The 2010 estimated total number of HAIs attributed to the three pathogens was 1.325 million and an economic burden attributable to the pathogens of $13.1 billion. There were 1.046 million MRSA HAIs, 219,000 C.Diff HAIs, and 59,000 VRE HAIs. Sensitivity analysis of the incidence rate revealed that the total number of HAIs ranged from 844,000 to 1.81 million and the associated economic burden ranged from $8.46 billion to $17.73 billion. Sensitivity analysis of the costs ranged from $9.8 billion to $16.4 billion.

## Conclusion

MRSA, C. Diff, and VRE result in significant HAIs and economic burden in patients hospitalized in US acute care hospitals. For hospitals to remain profitable, one important step should be a rigorous analysis of HAIs and comprehensive preventative programs to reduce their occurrence. Quality improvement programs to reduce preventable HAIs such as MRSA, C. Diff, and VRE will not only improve patient quality, but will increase hospital profitability by reducing HAIs that are subject to financial disincentives.

## Disclosure of interest

A. Mitchell Employee of Johnson & Johnson, R. Rohde: None declared, P. Mallow Consultant for Johnson & Johnson, D. Buskirk Employee of Johnson & Johnson
